# Protonophore treatment augments energy expenditure in mice housed at thermoneutrality

**DOI:** 10.3389/fphys.2024.1452986

**Published:** 2024-09-24

**Authors:** Daniel G. Sadler, Reid D. Landes, Lillie Treas, James Sikes, Craig Porter

**Affiliations:** ^1^ Arkansas Children’s Nutrition Center, Little Rock, AR, United States; ^2^ Arkansas Children’s Research Institute, Little Rock, AR, United States; ^3^ Department of Pediatrics, Univesrity of Arkansas for Medical Sciences, Little Rock, AR, United States; ^4^ Departments of Biostatistics, University of Arkansas for Medical Sciences, Little Rock, AR, United States

**Keywords:** protonophores, energy expenditure, thermoneutral, mitochondria, mouse models

## Abstract

**Background:**

Sub-thermoneutral housing increases facultative thermogenesis in mice, which may mask the pre-clinical efficacy of anti-obesity strategies that target energy expenditure (EE). Here, we quantified the impact of protonophore treatment on whole-body energetics in mice housed at 30°C.

**Methods:**

C57BL/6J mice (*n* = 48, 24M/24F) were housed at 24°C for 2 weeks; 32 (16M/16F) were then transitioned to 30°C for a further 4 weeks. Following 2 weeks acclimation at 30°C, mice (*n* = 16 per group, 8M/8F) received either normal (0 mg/L; Control) or supplemented (400 mg/L; 2,4-Dinitrophenol [DNP]) drinking water. Mice were singly housed in metabolic cages to determine total EE (TEE) and its components via respiratory gas exchange. Mitochondrial respiratory function of permeabilized liver tissue was determined by high-resolution respirometry.

**Results:**

Transitioning mice from 24°C to 30°C reduced TEE and basal EE (BEE) by 16% and 41%, respectively (both *P *< 0.001). Compared to 30°C controls, TEE was 2.6 kcal/day greater in DNP-treated mice (95% CI: 1.6–3.6 kcal/day, *P *< 0.001), which was partly due to a 1.2 kcal/day higher BEE in DNP-treated mice (95% CI: 0.6–1.7 kcal/day, *P *< 0.001). The absolute TEE of 30°C DNP-treated mice was lower than that of mice housed at 24°C in the absence of DNP (DNP: 9.4 ± 0.7 kcal/day vs. 24°C control: 10.4 ± 1.5 kcal/day). DNP treatment reduced overall body fat of females by 2.9 percentage points versus sex-matched controls (95% CI: 1.3%–4.5%, *P *< 0.001), which was at least partly due to a reduction in inguinal white fat mass.

**Conclusion:**

Protonophore treatment markedly increases EE in mice housed at 30°C. The magnitude of change in TEE of mice receiving protonophore treatment at 30°C was smaller than that brought about by transitioning mice from 24°C to 30°C, emphasizing that housing temperature must be considered when assessing anti-obesity strategies that target EE in mice.

## Introduction

The obesity pandemic affects more than 4 in 10 adults in the United States. Upward of $150 billion is spent annually to treat acute and chronic conditions related to obesity (Finkelstein et al., 2009). Despite this, there are very few drugs approved for obesity treatment. Currently available pharmacotherapy regimens rarely achieve weight loss of greater than 10% and often fail to provide patients with long-term weight control (Calderon et al., 2022; Rucker et al., 2007). The limited availability of effective drugs that target obesity is likely explained by the complex multifaceted nature of the disease. Indeed, until the recent development of the GLP1 agonist Semaglutide, only bariatric surgery was capable of bringing about significant and sustained weight loss in individuals with obesity.

The paucity of anti-obesity drugs may also be related to the failure of some pre-clinical obesity models to accurately recapitulate human obesity. Indeed, laboratory mice housed under standard vivarium temperatures (20°C–26°C) are relatively resistant to diet-induced obesity (Feldmann et al., 2009; Keipert et al., 2020; Liu et al., 2003) and exhibit a total-to-basal energy expenditure (EE) ratio 2–3 times greater than that of humans (Fischer et al., 2018; Keijer et al., 2019). This reflects the fact that sub-thermoneutral housing temperatures impose thermal stress on mice that can induce significant non-shivering thermogenesis (Fischer et al., 2018; Keijer et al., 2019; Škop et al., 2020) and changes in the proteomic signatures of adipose tissue depots (Sadler et al., 2022). Accordingly, the outcome(s) of rodent studies investigating anti-obesity therapies may be influenced by housing temperature.

Protonophores have long been considered anti-obesity agents given their ability to potently uncouple mitochondria. The protonophore 2,4-dinitrophenol (DNP) was known to augment EE as early as the 1890’s (Dunlop, 1934), which resulted in its use as a weight loss drug in the 1930’s (Tainter et al., 1934). However, DNP was withdrawn from patient use due to unfavorable side effects (Grundlingh et al., 2011). Despite this, mitochondrial uncoupling through DNP treatment has been demonstrated to improve glucose tolerance and reduce adiposity without causing toxicity in pre-clinical models of obesity (Goldgof et al., 2014; Perry et al., 2013). Further studies have demonstrated improved metabolic health with alternative protonophores (Alexopoulos et al., 2020; Kalinovich et al., 2005; Tao et al., 2014), renewing interests in the anti-obesity effects of mitochondrial uncouplers.

Given the renewed interest in the anti-obesity potential of drugs such as protonophores, it is important to study the efficacy and safety of these compounds using preclinical models that are not already hypermetabolic. Indeed, DNP’s ability to increase EE and lower adiposity in mice at 30°C is diminished at 22°C (Goldgof et al., 2014). However, there is limited data available on how protonophores impact total energy expenditure (TEE) and its components in male and female mice housed at thermoneutrality. To this end, we investigated the impact of housing temperature and protonophore treatment on whole-body energetics in male and female mice.

## Methods

### Animals

Male and female C57Bl/6J (#000664, Jackson Laboratories, Bar Harbor, ME, United States) mice (6–8 weeks old) were individually housed at 24°C on a standard light cycle (light 7am–7pm) with *ad libitum* access to a standard chow diet (TD.95092 [18.8% protein, 17.2% kcal fat, 63.9% kcal carbohydrate, 3.8 kcal/G] Envigo Teklad Diets, Madison, WI, United States). After ∼2 weeks of acclimation, one group of animals were maintained at 24°C (*n* = 16) and the remaining two groups were transitioned to 30°C (*n* = 32) for a total of 4 weeks. After 2 weeks of acclimation at the assigned housing temperature, the two groups of mice (*n* = 16 per group, 8 male/8 female) housed at 30°C were further randomized to receive either normal drinking water (0 mg/L; Control) or DNP-supplemented (400 mg/L; DNP) drinking water for a further 2 weeks. Animal weights and body composition were recorded before and after 2 weeks of DNP treatment. At conclusion of the study, all animals were euthanized in a rising concentration of CO_2_, and tissue samples were collected. This protocol was approved by the Institutional Animal Care and Use Committee at the University of Arkansas for Medical Sciences. The experimental approaches used in preliminary studies to determine the DNP dose (400 mg/L) used in the current study are described in the Supplementary Material. Due to the nature of the current studies (i.e., housing temperature changes and administration of a hazardous compound via drinking water) research staff were not blinded to group allocation. Whenever possible, the collation and analysis of raw data was performed by researchers that were blinded to group allocation.

### Body composition analysis

Body composition was measured by quantitative magnetic resonance imaging (qMRI) using the EchoMRI-1100 (EchoMRI, Houston, Texas, United States). Fat free mass (FFM) was calculated as the difference between body weight and fat mass (FM).

### Metabolic and activity phenotyping

From days 12–16 and 37–41 of the 6-week study, all mice underwent metabolic and activity phenotyping. Mice were individually housed for 5-6 consecutive days in specialized cages that allowed rates of oxygen consumption (VO_2_) and carbon dioxide production (VCO_2_) to be continuously measured to calculate total energy expenditure and its components (Sable Systems International, Las Vegas, NV, United States). During this time, food and water intake, activity, and voluntary wheel running were also continuously recorded. Animals were housed in environmental cabinets to control ambient temperature for metabolic phenotyping experiments. For mice transitioned from 24°C to 30°C, cabinet temperature was increased from 24°C to 30°C by 1°C per hour on day 3 of metabolic data collection (days 12–16 of the study).

Data files from metabolic phenotyping experiments were processed using macros to distill data into hourly averages/totals and provided circadian reports for each 12-h light cycle (Sable Systems International, Las Vegas, NV, United States). Rates of EE were calculated from VO_2_ and VCO_2_ using the Weir equation:

$$EE\;\left(\frac{kcal}{hr}\right)=60\times \left(0.003941\times {VO}_{2}\right)+\left(0.001106\times {VCO}_{2}\right)$$

TEE was calculated by summing average rates of EE for the light and dark cycle (kcal/cycle). Basal EE (BEE) was calculated from the 30-min period with the lowest average EE (kcal/hour) in the light cycle and extrapolated to 24 h. Resting EE (REE) was calculated from the 30-min activity period with the lowest average EE (kcal/hour) in the light cycle and extrapolated to 24 h. Energy expenditure of individual wheel running bouts were calculated as the EE exceeding the BEE associated with each individual running bout. Total daily wheel EE was calculated by summing the EE of all wheel running bouts for each day and averaging across the measurement period. Peak activity EE (AEE) was calculated as the highest EE (kcal/hour) over a 15-min period in the dark cycle. All data were averaged over two consecutive days. Daily energy intake was calculated by summing the total food consumed by the mouse during the experimental period and multiplying that sum by the diet energy density, before dividing by the number of days in the experimental period. All meters were the measurement of cage ambulation that included all gross and fine movements but excluded any meters ran directly on the running wheel. Wheel meters for two male (24°C) and one female (30°C), and all meters for one male (30°C + DNP) and one female (30°C) were excluded due to technical issues pertaining to the collection of running wheel use data.

### Tissue collection

Mice were euthanized in a rising concentration of CO_2_. Subsequently, liver, interscapular brown adipose tissue (iBAT) and inguinal white adipose tissue (iWAT) depots were excised and weighed. Liver tissue was then immediately placed in ice-cold BIOPS preservation buffer (10 mM Ca-EGTA buffer, 0.1 µM free calcium, 20 mM imidazole, 20 mM taurine, 50 mM K-MES, 0.5 mM DTT, 6.56 mM MgCl_2_, 5.77 mM ATP, 15 mM phosphocreatine, pH 7.1) for respirometry measures. Remaining tissue was frozen and stored at −80°C for further analyses.

### High resolution respirometry

Liver tissue was stored in ice-cold BIOPS preservation buffer from the time of sample collection until it was analyzed by high resolution respirometry. Liver tissue was minced into in BIOPS buffer before being blotted on filter paper briefly prior to being weighed. Approximately 1–2 mg (wet weight) of liver was transferred into the chamber of an Oxygraph-2K (O2k) high-resolution respirometer (Oroboros Instruments, Innsbruck, Austria) containing 2 mL of buffer (MiR05 composition: 0.5 mM EGTA; 3 mM MgCl2; 0.5 M K–lactobionate; 20 mM taurine; 10 mM KH_2_PO_4_; 20 mM HEPES; 110 mM sucrose; and 1 mg/mL essential fatty acid free bovine serum albumin) for assessment of mitochondrial bioenergetics. High resolution respirometry analysis was performed on the same day of sample collection, typically within 2–4 h of euthanasia. Temperature was maintained at 37°C and O_2_ concentration within the range of 200–400 nmol/mL for all respirometry analyses. O_2_ concentration within the Oxygraph chamber was recorded at 2–4 s intervals (DatLab, Oroboros Instruments, Innsbruck, Austria) and used to calculate respiration per milligram of wet tissue weight.

Upon loading into the chamber, liver was permeabilized by the addition of digitonin (2 μM). Mitochondrial respiration in the leak state was subsequently assayed following the addition of saturating concentrations of substrates (20 mM pyruvate, 2 mM malate, 10 mM glutamate and 20 mM succinate). Thereafter, saturating concentrations of ADP (7.5 mM) and oligomycin (OMY; 15 μM) were added to determine maximal respiration rates linked to ATP production (OXPHOS) and proton leak, respectively. Finally, carbonyl cyanide m-chlorophenylhydrazone (CCCP; 20 μM) was titrated into the chamber to determine maximal electron transfer capacity. Where measured electron transfer capacity was lower than OXPHOS, values of OXPHOS were used in place of electron transfer capacity. Respiratory (RCR) and flux (FCR) control ratios were calculated from respirometry data as qualitative indices of mitochondrial respiratory function.

### Statistical analysis

Outcomes from individual animals are presented in the figures and are summarized with the groups’ means and standard deviations (SDs). When evaluating the effects of transitioning mice from 24°C to 30°C, the outcomes were analyzed with a repeated measures ANOVA having sex, time, and their interaction as factors. There was evidence that the variability differed between pre- and post-transition for all outcomes examined; hence the within-animal covariance matrix was assumed to be general. The primary comparison of interest was between pre- and post-transition (the time main effect). When there was evidence of a time× sex interaction at the *P *< 0.20 level, then the pre- vs. post-transition comparison was made within each sex, and the results adjusted for these 2 comparisons with Bonferroni’s method.

When evaluating DNP effects, the outcomes were analyzed with a two-factor ANOVA having DNP administration, sex, and their interaction as factors. Normal and constant variance assumptions were checked. Normal assumptions were violated for only one outcome (body mass). For this one outcome, we performed a nonparametric analysis to determine whether the inferences based on the normal assumptions differed. Constant variance assumptions were violated for several outcomes. In those instances, we allowed variances among select groups to differ, and we estimated the error of degrees of freedom with the Kenward-Roger method (Kenward and Roger, 2009). Our primary comparison of interest was the main effect of DNP. If, however, the DNP sex interaction was significant at the *P* < 0.20 level, we present the sex-specific DNP effects, and adjust these comparisons with Bonferroni’s method. For significant sex-dependent results, we report the interaction effect, which compares the DNP effect in females to that in males. Our significance level was 0.05. The models described above were fitted with the MIXED procedure in SAS/STAT^©^ version 9.4 (SAS Institute, Cary, NC). Though outcomes from the control animals housed continuously at 24°C are included in the figures, these data were not included in the statistical models; they are for visual comparison purposes only. The data and software code producing the results are provided in the Supplementary Material.

### Sample size considerations

We wanted to be able to detect a difference in means of total energy expenditure between DNP-treated and control animals of size 1.4 SD, where SD is assumed constant between populations. The sample size was calculated for detecting a main effect of DNP with 0.90 power on a 0.01 level test in the two-factor ANOVA described above. The DNP group size was calculated to be 16 (8 males); thus 32 total animals that were to be transitioned to 30°C. We also used *n* = 16 (8 males) for the control animals continuously housed at 24°C. Altogether we used 48 animals to evaluate transition and DNP effects.

## Results

### Dose-response to DNP treatment

To determine the optimal concentrations of DNP to administer in this study, a pilot study was performed where mice received DNP-supplemented drinking water ranging from 0 to 1,400 mg/L (in 200 mg/L increments, *n* = 4 mice per group) at 24°C. Concentrations of DNP >400 mg/L acutely attenuated mouse TEE (despite increased BEE), which was the result of reduced food and water intake, as well as a marked reduction in wheel running (Supplementary Figure S1). In contrast, a dose of 400 mg/L did not markedly affect animal behavior but did augment BEE and TEE.

### Transitioning mice from 24°C to 30°C lowers energy expenditure

**FIGURE 1:**
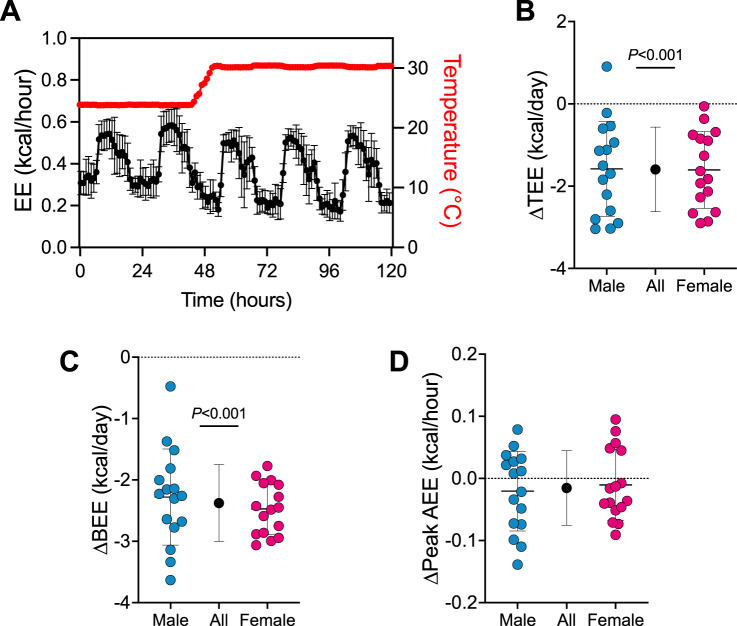
Energy expenditure rapidly changes upon the transition from 24°C to 30°C. **(A)** Representative energy expenditure trace upon the transition from 24°C to 30°C housing (*n* = 32). Change in **(B)** TEE, **(C)** BEE and **(D)** peak AEE from pre- to post- transition from 24°C to 30°C. Blue and red circles represent male and female animals (*n* = 16), respectively. Black points represent group means when male and female data combined. Data are mean ± SD.

First, we determined how the transition from 24°C to 30°C affects TEE and its components (see Figure 1; Supplementary Figure S2). A trace of EE during the transition from 24°C to 30°C is presented in Figure 1A. Transitioning mice from 24°C to 30°C reduced TEE by 1.6 kcal/day (95% CI: 1.2–2.0 kcal/day, *P *< 0.001; Figure 1B) and BEE by 2.4 kcal/day (95% CI: 2.1–2.6 kcal/day, *P *< 0.001; Figure 1C). Peak AEE was similar in all mice pre- and post-temperature transition (Figure 1D). There was no evidence that transition effects depended on sex (all 3 time×sex interactions *P* > 0.390).

### DNP treatment augments energy expenditure in male and female mice housed at 30°C

**FIGURE 2:**
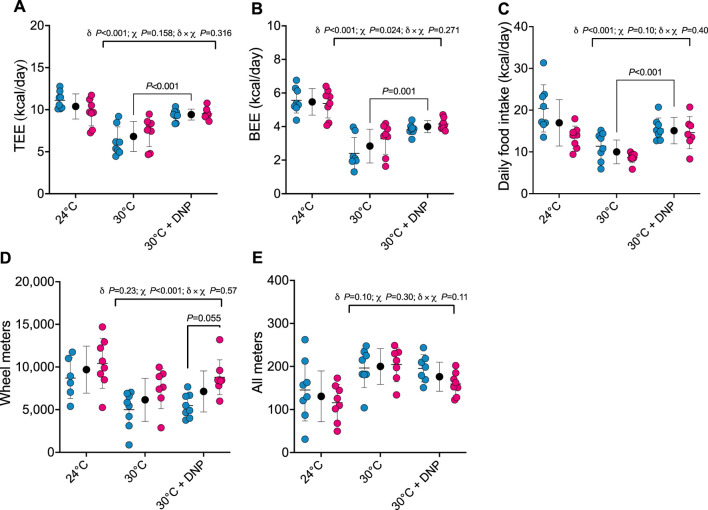
DNP treatment augments energy expenditure. **(A)** TEE. **(B)** BEE. **(C)** daily food intake. **(D)** Wheel meters. **(E)** all meters. Blue, red, and black circles represent males, females and males/female combined, respectively. Data are values ± SD (*n* = 8 per group; total *n* = 48). δ main effect of DNP treatment. χ main effect sex. δ × χ DNP and sex interaction. Protonophore treatment alters body composition of mice housed at thermoneutrality in a sex-dependent manner.

After establishing the impact of transitioning mice from 24°C to 30°C on TEE and its components, we assessed how DNP affects these parameters in mice acclimated to 30°C. TEE was 2.6 kcal/day greater in all DNP-treated mice (95% CI: 1.6–3.6 kcal/day, *P *< 0.001) when compared to all 30°C control mice (Figure 2A). Of interest, though TEE in mice treated with DNP was significantly higher than the 30°C control mice, their mean was still lower than that of mice housed at 24°C in the absence of DNP (DNP: 9.4 ± 0.7 kcal/day vs. 24°C control: 10.4 ± 1.5 kcal/day). BEE was 1.2 kcal/day greater in DNP-treated mice (95% CI: 0.6–1.7 kcal/day, *P* < 0.001) when compared to the 30°C control mice (Figure 2B). Daily food intake of DNP-treated mice was 5.1 kcal/day higher than controls (95% CI: 2.8–7.3 kcal/day, *P* < 0.001; Figure 2C). DNP treatment did not alter wheel running activity compared to 30°C control mice; though, females ran more than males averaged over both 30°C conditions (Figure 2D). For all meters (ambulation), there was some evidence that the DNP effect depended on sex (DNP×sex interaction *p* = 0.110). The 30°C DNP females moved 45 m/day less than the 30°C control females (95% CI: 0–90 m/day; Figure 2E), whereas there was little difference between the DNP and control males at 30°C.

**FIGURE 3:**
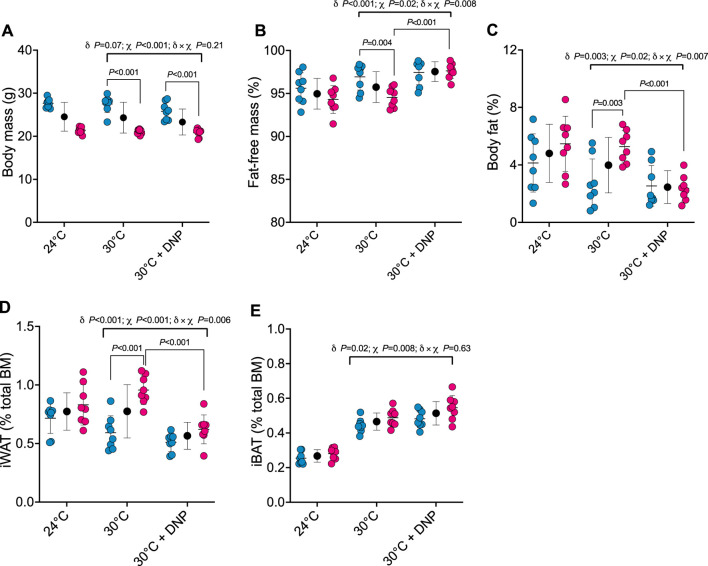
DNP treatment alters body composition in a sex-dependent manner. **(A)** Body mass. **(B)** Percent fat-free mass. **(C)** Percent body fat. **(D)** Relative iWAT mass. **(E)** Relative iBAT mass. Blue, red, and black circles represent males, females and males/female combined, respectively. Data are values ± SD (*n* = 8 per group; total *n* = 48). δ main effect of DNP treatment. χ main effect sex. δ × χ DNP and sex interaction.

Next, we determined how DNP affects body composition. Body mass of male and female mice was similar between DNP and 30°C control groups (Figure 3A). DNP effects on body composition were sex dependent (Figures 3B, C): DNP decreased relative fat mass in females by 2.9 percentage points (*P *< 0.001), and the female’s DNP-decrease was 2.7 percentage points more (95% CI: 0.8 to 4.7 percentage points, interaction *p* = 0.007, Figure 3C) than the non-significant DNP-decrease of 0.2 percentage points experienced by males. Likewise, DNP decreased relative iWAT mass by 0.34 percentage points in females (*P *< 0.001; Figure 3D), and the female’s DNP-decrease was 0.25 percentage points more (95% CI: 0.08 to 0.43 percentage points, interaction *p* = 0.006) than the non-significant DNP-decrease of 0.08 percentage points experienced by males (Figure 3D). Compared to 30°C control mice, DNP treatment increased iBAT mass by 0.05 percentage points (95% CI: 0.01 to 0.09, *p* = 0.017; Figure 3E); the DNP effect did not depend on sex (interaction *p* = 0.626).

### Protonophore treatments alters hepatic bioenergetics in a sex-dependent manner

**FIGURE 4:**
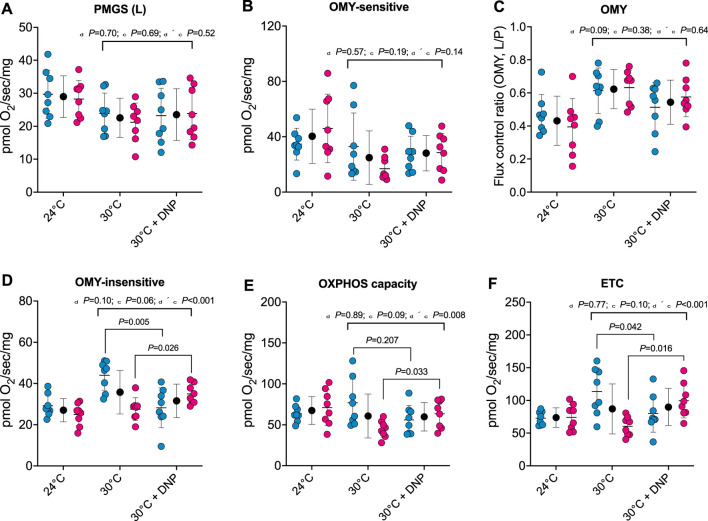
Protonophore treatment alters liver bioenergetics in a sex-dependent manner. Hepatic bioenergetics in response to substrates, uncoupler and inhibitors. **(A)** PGMS-driven leak respiration; **(B)** OMY-sensitive respiration; **(C)** Flux control ratio for respiration in the presence of OMY (L/P); **(D)** OMY-insensitive respiration; **(E)** OXPHOS capacity **(F)** Electron transfer capacity (ETC.). PGMS = Pyruvate/glutamate/malate/succinate. OMY = oligomycin. (L), leak state; **(E)**, electron transfer. Blue, red, and black circles represent males, females and males/female combined, respectively. Values are means ± SD (*n* = 8 per group; total *n* = 48). δ main effect of DNP treatment. χ main effect sex. δ × χ DNP and sex interaction.

To establish whether DNP alters the respiratory capacity and coupling control of hepatic mitochondria, we performed high-resolution respirometry on digitonin permeabilized liver tissue. No effects of DNP were found in PGMS-driven respiration, OMY-sensitive respiration, or OMY flux control (Figure 4A/B/C). However, DNP effects were present in OMY-insensitive respiration, OXPHOS, and ETC., and the DNP effects statistically differed between the sexes. For OMY-insensitive respiration, DNP increased O_2_ flux in females by 7.4 pmol/O_2_/mg (*p* = 0.026) and decreased O_2_ flux in males by 15.7 pmol/O_2_/mg (*p* = 0.005): thus a 23.1 pmol/O_2_/mg difference in DNP effects between the sexes (95% CI: 12.8, 33.4 pmol/O_2_/mg, *P *< 0.001, Figure 4D). For OXPHOS, DNP increased O_2_ flux in females by 19.0 pmol/O_2_/mg (*p* = 0.033) and decreased O_2_ flux in males by 20.9 pmol/O_2_/mg (*p* = 0.207): thus a 40.0 pmol/O_2_/mg difference in DNP effects between the sexes (95% CI: 11.2–68.8 pmol/O_2_/mg, *p* = 0.009, Figure 4E). Finally, for, ETC., DNP increased O_2_ flux in females by 39.2 pmol/O_2_/mg (*p* = 0.016) and decreased O_2_ flux in males by 33.5 pmol/O_2_/mg (*p* = 0.042): thus a 72.8 pmol/O_2_/mg difference in DNP effects between the sexes (95% CI: 33.0–112.6 pmol/O_2_/mg, *P *< 0.001, Figure 4F).

## Discussion

We investigated how housing temperature and protonophore treatment impact whole body EE and behavior in mice. We demonstrate that chronic protonophore treatment augments the TEE of mice housed at 30°C by augmenting BEE. Of interest, TEE of protonophore-treated mice housed at 30°C were still lower than those of mice housed at 24°C in the absence of protonophores. This emphasizes the impact of housing temperature on facultative thermogenesis in mice and highlights the need to consider housing temperature when investigating drugs that target EE.

It has long been known that protonophores such as DNP augment EE in humans (Dunlop, 1934). More recent work has demonstrated that DNP treatment elevates EE of mice housed at thermoneutrality, although this effect is lost in mice housed at 22°C (Goldgof et al., 2014). Similarly, we observed a robust increase in the basal, and therefore, TEE of male and female mice treated with DNP when housed at 30°C. These findings suggest that protonophores can potently augment murine TEE in the absence of cold-induced non-shivering thermogenesis. Interestingly, the magnitude of change in TEE we observed with DNP treatment at 30°C was smaller than the change evoked in the temperature transition from 24°C to 30°C. This highlights the importance of considering housing temperature when designing experiments to study drugs that target EE. Furthermore, our findings underscore the ability of protonophores to alter bioenergetics independent of cold-induced UCP1 mediated uncoupling (Bertholet et al., 2022).

Our finding that energy intake increases concurrently with EE following protonophore treatment supports the notion that mammalian energy intake is somewhat coupled to EE (Ono-Moore et al., 2020) – at least in the paradigm of a significant hypermetabolic response to protonophore treatment. However, this observation contrasts previous studies reporting unaltered energy intake and elevated EE in rodents following protonophore treatment (Goldgof et al., 2014; Alexopoulos et al., 2020; Axelrod et al., 2020). Perhaps, the discrepancies between these findings and those in the present study are due to the methods used to quantify animal energy intake–one study assessed energy intake biweekly in group housed animals, whereas we quantified daily energy intake in singly housed mice by real-time food intake measures, where animals also had access to running wheels. These discrepancies may also be explained by the use of high versus low-fat diets. Moreover, in the present study we did not directly measure dietary absorption efficiency, which could have been influenced by protonophore treatment. Since we observed reductions in body fat mass in females in response to protonophore treatment, despite no changes in total body mass, this suggests that despite a matching between TEE and energy intake, protonophore treatment altered body composition.

At thermoneutrality, brown adipose tissue activation is minimal in rodents (Fischer et al., 2018; Keijer et al., 2019). Given that mice were housed at approximately thermoneutrality (30°C) in this study, it is likely that other tissues contributed to the DNP-mediated increase in TEE. It has been suggested that the liver may be a key site of DNP-mediated mitochondrial uncoupling based on reported elevations in circulating β-hydroxybutyrate (Goldgof et al., 2014). Indeed, DNP treatment has been reported to increase state 4 respiration of isolated liver mitochondria (Perry et al., 2013). We demonstrated that noncoupled respiration of female, but not male livers were elevated in response to DNP, which might partly explain why DNP attenuated adiposity in only female mice. What explains this sex-specific bioenergetic response to protonophore treatment remains unclear. However, there is apparent sexual dimorphism in weight gain and adipose tissue mitochondrial uncoupling protein expression following a high-fat diet in mice (Norheim et al., 2019; MacCannell et al., 2021). One consideration for these findings is that liver tissue was minced and kept in an ice-cold preservation buffer for ∼1–2 h prior to analysis, where it is possible that residual DNP was removed from liver tissue. Nevertheless, mitochondria from livers of both control and DNP-treated mice responded robustly to protonophore titration, suggesting that liver mitochondria are likely more uncoupled *in-vivo* in protonophore treated mice.

Another key observation of the current study was that liver bioenergetics exhibited sexual dimorphism. We observed greater hepatic respiratory capacity in both the coupled and uncoupled state in males versus females in the absence of DNP. These data are in contrast to previous studies reporting greater hepatic maximal oxidative capacity and maximal electron transfer capacity in female versus male rodents (Justo et al., 2005; Schulze et al., 2018; Valle et al., 2007), although the experiments performed in these studies used isolated mitochondria, compared to permeabilized tissue in the current report. As previous studies have demonstrated, bioenergetic data interpretation may be influenced by whether one is studying isolated mitochondria or permeabilized tissue (Picard et al., 2010).

It is well known that DNP has a narrow therapeutic window. The 400 mg/L dose of DNP was chosen as an experimental dose here because of its ability to increase basal and TEE, which was not observed with doses >400 mg/L in an acute pilot study. Although we did not assess drug toxicity, it is unlikely our chosen dose caused acute toxicity given a previous report documenting 800 mg/L DNP (twice that of the present study) treatment did not affect plasma alanine aminotransferase levels or liver histology (Goldgof et al., 2014). In this study by Goldgof and colleagues, 800 mg/L daily DNP treatment of female C57BL/6J mice housed at 30°C caused mild weight reduction over 1–2 weeks and did not affect food intake (Goldgof et al., 2014). The greater dose used in this previous study likely contributed to reduced body mass–a finding not reproduced in this investigation. However, it should be noted that in the current study we provided mice with running wheels during DNP treatment. Given that mice voluntarily run ∼5–10 km daily when provided with wheels, running wheel provision can have a marked impact on energy balance experiments. In preliminary studies, escalating DNP doses (particularly >400 mg/L) significantly reduced voluntary wheeling running, to an extent that DNP doses of 600 mg/L or greater did not significantly increase TEE, despite having a marked impact on BEE. Thus, we chose a dose that had the least impact on running behavior while still augmenting REE (i.e., 400 mg/L). Consideration of baseline physical activity and it’s modulation by protonophore therapy may be useful in terms of providing a more holistic understanding of the impact of mitochondrial uncouplers on TEE.

Though some doses of DNP may be tolerable and acutely non-toxic in rodents, DNP is still unsuitable for the treatment of human obesity (Grundlingh et al., 2011). Nevertheless, the data in the present study underscore the utility of protonophores as agents that induce hypermetabolism, supporting a role for protonophores as potential treatments for obesity. Recent advancements involving DNP analogues and alternative protonophores with improved safety profiles show promise in animal models of obesity (Perry et al., 2013; Alexopoulos et al., 2020; Axelrod et al., 2020). Our data emphasize the importance of assessing the preclinical safety and efficacy of such compounds in rodents housed at temperatures that thwart facultative thermogenesis, while also taking into consideration other important variables such as biological sex and physical activity levels.

Overall, we have demonstrated that protonophore treatment augments TEE and, in a sex-dependent manner, reduces adiposity in mice housed at 30°C. Together, these findings underscore the efficacy of compounds that uncouple mitochondria–such as protonophores–in the context of augmenting energy expenditure. However, our current data highlight the critical importance of studying rodents at appropriate housing temperatures when assessing the impact of compounds that target energy metabolism.

## Data Availability

The datasets presented in this study can be found in online repositories. The names of the repository/repositories and accession number(s) can be found below: https://doi.org/10.6084/m9.figshare.20397468.
