# Supplementary material for: Protonophore treatment augments energy expenditure in mice housed at thermoneutrality
Source: Front Physiol. 2024 Sep 24;15:1452986. doi: 10.3389/fphys.2024.1452986 (PMC11458463; doi:10.3389/fphys.2024.1452986)
Supplement: Supplementary file 1 [file Image1.pdf]

Supplementary Information

Supplementary Methods

Male C57Bl/6J (#000664, Jackson Laboratories, Bar Harbor, ME, USA) mice (6-8 weeks old) were individually housed at 24°C on a reverse light cycle (light 7pm – 7am) with ad libitum access to a standard chow diet (TD.95092 [18.8% protein, 17.2% kcal fat, 63.9% kcal carbohydrate, 3.8 Kcal/gram)] Envigo Teklad Diets, Madison, WI, USA). After ~1 week of acclimation, animals were randomly assigned to receive either normal drinking water (0 mg/L; CTRL) or DNP-supplemented (200-1400 mg/L; DNP) drinking water. Animals received either 0, 200, 400, 600, 800, 1000, 1200 or 1400 mg/L DNP (n=4 per dose). Animal weights and body composition were monitored prior to and following 7 days of assigned treatment. If two or more mice in any dose group met criteria for moderate pain/distress (up to a 50% reduction in feeding and/or a 20% reduction in body mass) then the DNP dose was defined as not being tolerable. Metabolic and activity phenotyping was performed over 10 days using Promethion metabolic cages, as outlined in the main manuscript. At the conclusion of the study, all animals were euthanized in a rising concentration of CO_2. This protocol was approved by the Institutional Animal Care and Use Committee at the University of Arkansas for Medical Sciences.

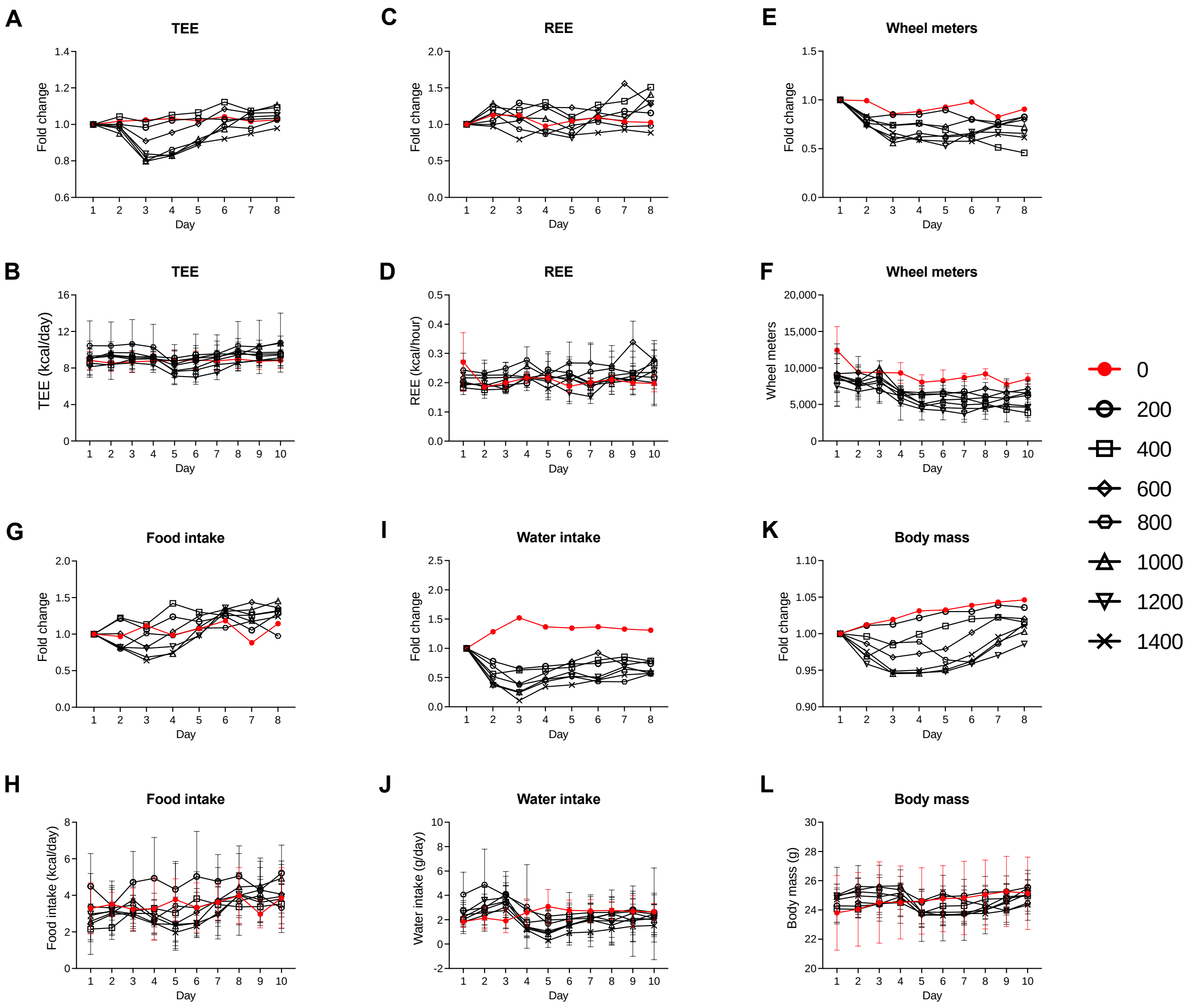

Supplementary Figure 1. Dose responses to DNP. Total energy expenditure (A) fold-changes and (B) absolute rates. Resting energy expenditure (C) fold changes and (D) absolute rates. Wheel meter (E) fold changes and (F) absolute data. Food intake as (G) fold changes and (H) absolute data. Water intake as fold changes (I) and (J) absolute values. Body mass (K) fold-change and (L) absolute values. Mice were housed at 24°C. Data are mean±SD (n=4 per group).

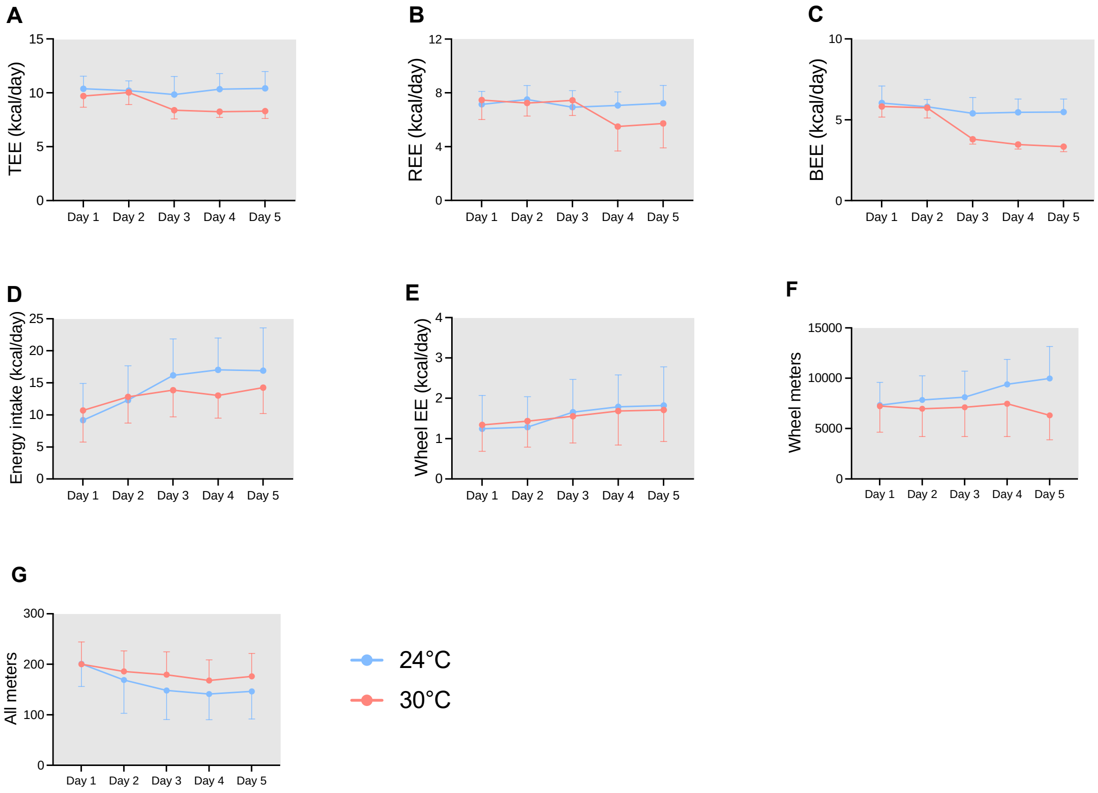

Supplementary Figure 2. Transitioning mice from 24°C to 30°C lowers energy expenditure. Daily measurements of (A) TEE, (B) REE, (C) BEE, (D) energy intake, (E) wheel EE, (F) wheel meters and (G) all meters in mice housed at 24°C for 5 days (24°C, *n*=16 (50% male) - blue trace) or 24°C for two days followed by a transition to 30°C for a further 3 days (30°C, *n*=32 [50% male] – red trace). Data are daily group means±SD.

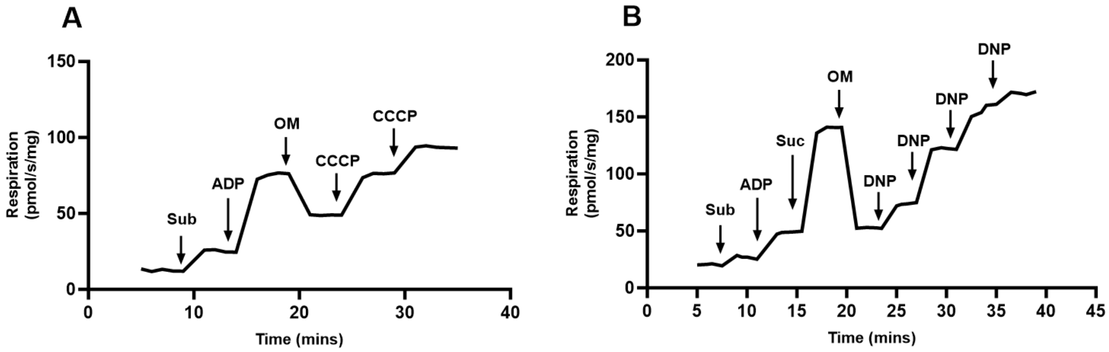

Supplementary Figure 3. High Resolution Respirometry Traces. (A) Representative oxygraph trace for experiments in Figure 4 where substrates (Sub: Pyruvate, malate, glutamate and succinate) were added to the chamber followed by ADP, oligomycin and 1µM CCCP titrations. (B) A representative oxygraph trace following addition of substrates (Sub: Pyruvate, malate and glutamate) to the chamber followed by, succinate, ADP, oligomycin and 5-10 µM DNP titrations. Traces in both A and B are permeabilized liver tissue.
